# EPIGENETICS OF STRESS AND ADAPTATION ACROSS HEALTHSPAN AND LIFESPAN

**DOI:** 10.1093/geroni/igz038.2693

**Published:** 2019-11-08

**Authors:** Luigi Ferrucci, Shabnam Salimi, Luigi Ferrucci

**Affiliations:** 1 Translational Gerontology Branch, Intramural Research Program, National Institute on Aging, National Institutes of Health, Baltimore, Maryland, United States; 2 University of Maryland Baltimore School of Medicine, Baltimore, Maryland, United States

## Abstract

Any stimulus that endangers body integrity (stressor) results in an adaptive response to resolve stressful state and determine adaptive or and maladaptive responses. Both chronic extrinsic and intrinsic stressors can produce long-lasting, epigenetic changes in various organs that can eventually result in accelerated changes in bio-physio-pathology. There is initial evidence that stress response involves mechanisms of the epigenetic basis of adaptation and stress response to biological aging and chronic diseases. With aging, homeostasis stability declines causing augmented vulnerability to the external and internal stress. Individuals in whom vulnerability trespass a certain threshold experience accelerated aging and deterioration of health and/ or “secondary aging” phenomena such as premature mortality. Because of substantial heterogeneity of the rate of decline in homeostatic stability, there is inter-individual variability in the age of appearance of chronic diseases and the increased risk of disability and mortality. Thus, tools for the quantification of a stress response would be clinically valuable. Therefore, this symposium suggests approaches to study the epigenetic basis of molecular adaptations across various age, organs’ health-span, and life-span.

